# *Saururus chinensis-*controlled allergic pulmonary disease through NF-κB/COX-2 and PGE_2_ pathways

**DOI:** 10.7717/peerj.10043

**Published:** 2020-09-24

**Authors:** MiKyung Song, Soon-Young Lee, Minhee Kim, Sangwoug Park, Juyeon Park, Yongbum Kwon, Dae-Hun Park

**Affiliations:** 1 Bio Technology R&D Center, WiLab Co., Ltd., Seoul, South Korea; 2 Department of Nursing, Dongshin University, Naju, South Korea; 3 Department of Forestry, Chonnam National University, Gwangji, South Korea

**Keywords:** *Saururus chinensis*, Allergic pulmonary disease, NF-kB/COX-2, PGE2, HPLC

## Abstract

*Saururus chinensis* is a perennial herb found in the northeastern regions of Asia, including Korea, China, and Japan, and is used in traditional medicine. Studies have identified the four major constituents in *Saururus chinensis* water extract (LHF618^®^) as miquelianin (11.75 ± 0.092 mg/g), rutin (1.20 ± 0.008 mg/g), quercitrin (2.38 ± 0.389 mg/g), and quercetin (0.068 ± 0.017 mg/g). *Saururus chinensis* can improve the symptoms of ovalbumin- or fine dust-induced allergic pulmonary disease by suppressing the effects of WBCs and neutrophils in BALF and IgE in the serum. *Saururus chinensis* dose-dependently recovered morphological changes such as mucous hyper secretion (from 2.7 ± 0.46 to 0.6 ± 0.65), pulmonary epithelial cell hyperplasia (from 2.4 ± 0.55 to 0.7 ± 0.67), and inflammatory cell infiltration (from 2.3 ± 0.45 to 0.6 ± 0.43), and effectively controlled cDNA levels and protein levels of IL-13. It inhibited NF-κB translocation and COX-2 protein synthesis and suppressed the expression of PGE_2_. Our results show that *Saururus chinensis* controlled allergic pulmonary disease via the anti-inflammatory pathways, NF-κB/COX-2 and PGE_2_. *Saururus chinensis* may be a promising drug candidate against fine dust-induced allergic pulmonary disease.

## Introduction

Allergic rhinitis and asthma are respiratory system diseases classified as allergic pulmonary diseases. Allergic rhinitis is an allergic pulmonary disease associated with the upper airway and its typical symptoms include nasal itching and discharge, upper airway obstruction, and watery eyes caused by allergens including pollens, mold, and dander (Wallace et al., 2008). Asthma is an allergic respiratory disease affecting the bronchioaleveolar region of the lower respiratory system and symptoms include mucous hypersecretion, pulmonary epithelial cell hyperplasia, inflammatory cell infiltration, and airway remodeling induced by allergens including pollen, mold, dander, house mite dust, pollutants, and temperature changes (Fehrenbach, Wagner & Wegmann, 2017; Hirota & Martin, 2013). Although asthma and allergic rhinitis can be classified as different diseases, they are highly similar and the term “allergy rhinobronchitis” has been suggested to link the diseases. The co-morbidity of the nose and bronchus is incredibly high with 75% of asthma patients affected by rhinitis and 20% to 40% of rhinitis patients affected by asthma (Palma-Carlos, Branco-Ferreira & Palma-Carlos, 2001).

Allergic rhinitis, asthma, and eczema are frequently diagnosed allergic disorders that may occur when subjects are repeatedly exposed to an allergen. These disorders may lead to allergic inflammation (Galli, Tsai & Piliponsky, 2008; Ferreira, 2004). It is important to control inflammation in allergy patients and the NK-κB/COX-2 and prostaglandin E_2_ (PGE_2_) axes are the primary pathways of inflammation (Mishra, Banga & Silveyra, 2018a; Mishra, Banga & Silveyra, 2018b). Many cytokines are closely associated with inflammation (Feghali & Wright, 1997), including interleukin-13 (IL-13), which causes chronic inflammation by stimulating B lymphocytes (Punnonen et al., 1993) and inducing inflammation, mucous hypersecretion, and fibrosis (Zhu et al., 1999).

Allergic pulmonary disease is incurable and combination therapies of corticosteroid hormones, leukotriene antagonists, and/or β_2_ agonists (Mishra, Yao & Levine, 2013) have been used to suppress its symptoms. However, this therapeutic method has adverse effects including growth suppression, hypertension, osteoporosis, gastric ulcers, and neurotoxicity (Wise, 2014; Ciriaco et al., 2013).

*Saururus chinensis* (S. chinensis, SC), a perennial herb found in the northeastern regions of Asia including Korea, China, and Japan, has been used as a traditional medicine for diseases such as edema, jaundice, gonorrhea, and other inflammatory diseases (Chung & Shin, 1990). Recent studies have reported the therapeutic effects of SC to include anti-inflammation (Kim et al., 2003; Cho, Cho & Song, 2005), antihypertension, vasodilation (Ryu et al., 2008), and anti-angiogenesis effects (Yoo et al., 2008), as well as the suppression of atopic dermatitis (Choi et al., 2008). However, there are few mechanistic studies on the active and therapeutic constituents of this botanical.

We investigated the co-morbidity of the nose and bronchus and the effects of SC on allergic rhinitis and asthma. We determined the biological constituents of SC and extracted the compounds that suppressed the symptoms of allergic pulmonary disease.

## Materials & Methods

### Plant Materials

Dried SC leaves (LHF618^^®^^) were purchased from Donggwang Pharmaceutical Company (Seoul, Korea), and visually identified by the Korea Pharmaceutical Trading Association (Seoul, Korea). A voucher SC specimen was deposited at the WiLab Co. Ltd. (Seoul, Korea). SC samples were prepared as follows: samples were soaked in water at 95 ± 2 °C for 4 h (1 kg/15 L); the resulting water was filtered, concentrated in a water bath under a vacuum using a rotary evaporator, and then spray-dried (SD) with 25% dextrin.

### Chemicals and reagents

All reagents were of analytical grade. Methanol and acetonitrile (ACN) were purchased from JT Baker (Phillipsburg, NJ, USA). Miquelianin, rutin, and quercetin were purchased from Sigma-Aldrich (St. Louis, MO, USA). Quercitrin was purchased from InterPharm (Koyang-si, Korea). Formic acid was purchased from Daejung Chemicals & Metals Co. Ltd. (Kyunggi, Korea) and fine dust (FD, Phyllite powder, PH-DG2000) was bought from Hongik Bio Tech Co. Ltd. (Jeonnam, Korea).

### HPLC analysis of SC

We used a Waters HPLC system with a Waters alliance e2695 and a Waters 2998 photodiode array detector. The Fortis C18 analytical column (150 × 4.6 mm, 5 µm pore size) was tested and filled with the same stationary phase. Samples A (0.01% Formic acid) and B (ACN) were used as the mobile phase under gradient conditions to analyze the samples. The mobile phase was filtered by vacuum through a 0.45 µm membrane filter. Chromatography was conducted on a gradient using a flow rate of 1.0 ml/min at 30 °C and was detected at various UV wavelengths for individual standards and SC samples. The SC extract powder was dissolved in 50% MeOH at 10 mg/ml. The chromatograms were processed using Empower 2 software, build 1154 (Waters, Milford, MA, USA).

### Animal schedule

We followed the protocols of our previous study for animal testing (Lee et al., 2019). Animal euthanasia guidelines were established prior to the start of the experiment and the experimenters checked the physical condition of all animals at least twice daily. Any abnormal behaviors from an animal during the experiment warranted the euthanasia of the animal and a necropsy was performed. Euthanasia was administered by an overdose of Zoletil delivered via intraperitoneal injection (Virbac, Carros, France) to preserve the BALF or respiratory system (lung) of the animal and to evaluate the suppression effects against allergic pulmonary diseases of SC. 84 BALB/c mice were purchased from Samtako Korea (Osan, Korea) and were acclimated for seven days prior to the start of our study. The mice were supplied with food and water ad libitum at 23 ± 3 °C, 55 ± 15% relative humidity, and 12 hr light per day, with observations of their physical conditions both prior to and during the study period. The animals were divided into 6 groups: tap water treatment group (CON), ovalbumin (OVA, Sigma-Aldrich, St. Louis, MO, USA) and FD treatment group (OVA + FD), OVA + FM and dexamethasone treatment group (OVA + FD + DEX), and after OVA and FD treated SC administrated groups (50 mg/kg, 150 mg/kg, and 300 mg/kg). Animal experiments were conducted twice using the same methods.

**Figure 1:**
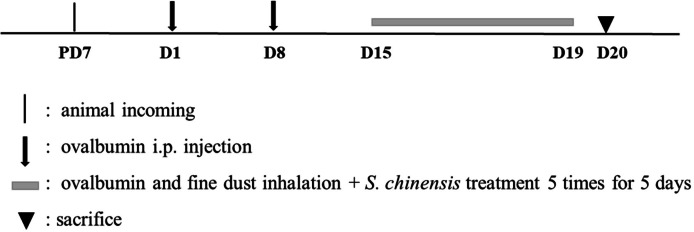
Experimental design for animal study.

All groups were treated twice at a one-week interval with intraperitoneal OVA, which consisted of 20 µg ovalbumin and 1 mg aluminum hydroxide hydrate (Sigma-Aldrich) in 500 µL saline, with the exception of the CON group. All mice were also exposed to a nebulizer mist (NE-U17, OMRON, Japan) of ovalbumin and fine dust for 30 min per day for 5 days, seven days after the second injection, except for the CON group. All groups were given 3 doses of an oral administration of tap water (CON or OVA + FD) or dexamethasone (OVA + FD + DEX) on the same exposure day but prior to the inhalation of ovalbumin and fine dust mist (Fig. 1).

### Ethics statement

Approval for the animal study was obtained from the Institutional Animal Care and Use Committee (Animal Study Approval No. CNU IACUC_YB-2019-48).

### Bronchoalveolar Fluid (BALF) and Serum Analysis

We applied the methods of our previous study (Lee et al., 2019) and used BALF and serum analysis to analyze the WBC, differential cell counts, and IgE levels. BALF was collected from the trachea of all of the test subjects using a feeding needle under anesthesia (50 mg/kg Zoletil intraperitoneal injection). BALF was gathered in a 0.4 mL phosphate-buffer saline and a differential cell count was conducted using a blood cell counter (Hemavet Multispecies Hematology System, Drew Scientific Inc, Waterbury, CT, USA). Diff-Quick stain (Thermo Fisher Scientific Inc., Pittsburgh, PA, USA) was performed (Skipper & DeStefano, 1989) after cytospining the BALF to evaluate the change of the immune cells. The serum IgE was measured by ELISA method (BD Bioscience, 555248, San Jose, CA, USA) to verify the modulation effect against allergies.

### Histopathological evaluation

We used a histopathological method of our previous study to assess the morphological changes caused by the chemical (OVA + FD) or therapeutic/preventive effects of SC (Lee et al., 2019). Lung samples from test subjects were fixed in 10% (V/V) formaldehyde, dehydrated in EtOH (99.9%, 90%, 80%, and 70%), and embedded in paraffin. Samples were sectioned at 4 µm thickness, and hematotoxylin & eosin (H&E) staining was performed with periodic acid schiff (PAS) to evaluate the morphological changes in the lung, including the glycoprotein changes. A microscope was used to obtain the images (Axioscope A1, Carl Zeiss, Gottingen, Germany) and the pathological level was scored from 0 (none) to 3 (severe) based on the representative pulmonary changes, including mucous hypersecretion (0, none; 1, little mucous releasing; 2, half packed mucous in whole duct; 3, packed mucous), epithelial cell hyperplasia (0, none; 1, corrugated wall; 2, folded epithelium; severe folded epithelium), and inflammatory cell infiltration (0,none; 1, few leukocytes; 2, moderate number of leukocytes; 3, large number of leukocytes).

### Reverse transverse-poly chain reaction (RT-PCR) Analysis

The mRNA levels of the AR-related cytokines were measured using the RT-PCR methods from our previous study, including IFN- γ, IL-5, IL-13, and TNF-α (Lee et al., 2019). The total RNAs were extracted from the lung tissue using an RNeasy Mini Kit (Qiagen, Hilden, Germany). 100 ng RNA was used for the reaction and the primers used are shown in Table 1. Denaturation occurred at 95 °C for 5 s and annealing/extension at 65 °C for 30 s for 40 cycles. The RT-PCR results were obtained using qTOWER2.2 (Analytik Jena, Jena, Germany).

**Table 1:** Primers sequences for RT-PCR.

**Genes**	**Primer Sequences**	
IFN-γ	Forward	5′-GGCCATCAGCAACAACATAAG-3′
	Reverse	5′-GTTGACCTCAAACTTGGCAATAC-3′
IL-5	Forward	5′-TGCATCAGGGTCTCAAGTATTC-3′
	Reverse	5′-GGATGCTAAGGTTGGGTATGT-3′
IL-13	Forward	5′-CAGCCCTCAGCCATGAAATA-3′
	Reverse	5′-CTTGAGTGTGTAACAGGCCATTCT-3′
TNF-α	Forward	5′-CTGAGTTCTGCAAAGGGAGAG-3′
	Reverse	5′-CCTCAGGGAAGAATCTGGAAAG-3′
GAPDH	Forward	5′-GTGGAGTCATACTGAACATGTAG-3′
	Reverse	5′-AATGGTGAAGGTCGGTGTG-3′

### Enzyme-linked immunosorbent Assay (ELISA)

ELISA was conducted following the methods of our previous study (Lee et al., 2019). Whole proteins were collected after cell lysis from the lung tissue using a buffer to measure the levels of proteins such as IFN-γ, IL-5, IL-13, and TNF-α. The buffer was made with a protease inhibitor and RIPA buffer (Thermo Fisher Scientific, Waltham, MA, USA). OptEIA mouse ELISAs (BD Biosciences, San Jose, CA, USA) were used to evaluate the protein levels of IFN-γ, IL-5, and TNF-α. IL-13 measurements were taken using the AbFrontier Cymax mouse ELISA kit (AbFronteir, Seoul, Korea) according to the manufacturer’s instructions. Whole proteins of identical weights from each group were homogenized with a lysis buffer and the supernatants were collected and analyzed with a microplate reader after centrifugation (MUTISKAN Sky, Thermo Fisher Scientific).

### Immunofluorescence assay

We used the immunofluorescent method as outlined in our previous study (Lee et al., 2019) to confirm the location of NF-κB in the nucleus or the cytoplasm, and the expression level of COX-2 in the cytoplasm. Paraffin-lung tissue samples were placed on slides and were incubated at room temperature for 1 hr with primary antibodies including NF-κB (PA5-16545, Thermo Fisher Scientific) and COX-2 (PA1-9032, Invitrogen, Carlsbad, CA, USA). Secondary antibodies such as FITC-conjugated IgG (315-095-003, Jackson Immunoresearch, West Grove, PA, USA) or Alexa Fluor 555-conjugated IgG (A-21127, Thermo Fisher Scientific) were used to counterstain and were used by DAPI (62249, Thermo Fisher Scientific). All images were acquired using a K1-Fluo confocal microscope (Nanoscope System, Daejeon, Korea) and the fluorescent intensity was analyzed by the software in the confocal microscope.

### Immunohistochemistry Assay –PGE2

Immunohistochemical analysis was conducted following the methods outlined in our previous study (Lee et al., 2019). Endogenous peroxidase was eliminated from tissue sections and antigens were retrieved using a sodium citrate buffer (0.1 M) for 10 min with 3% hydrogen peroxide in methanol. Non-specific antibody binding was blocked using normal serum and all samples prepared on slides were incubated for 1 h at 4 °C with primary antibodies (diluted 1:100, PGE2, bs-2639R, Bioss, MA, USA). All samples were incubated for 10 min to allow them to bind with secondary antibodies and the samples were reacted with a streptavidin peroxidase complex for 5 min (Vector Laboratories Universal Quick Kit, Burlingame, Canada). Chromogen solution was used to detect the signal 3,3-diaminobenzidine tetrahydrochloride substrate and the nucleuses were stained with Mayer’s hematoxylin. Images were obtained using an Axioscope A1 (Carl Zeiss) and the number of positive cells was determined by cell counts in 5 round areas of identical sizes for 3 immuno-stained samples per mouse.

### Statistical analysis

All data were described as mean ± standard deviation. Statistical consideration was significant at *p* < 0.05. The group difference was evaluated by one-way analysis of variance and Dunnett’s multiple comparison.

## Results and Discussion

### Constituents of SC leaf extracts

Reports have revealed that the effectiveness varies between the phytochemical components of ethanol and water extracts for anti-inflammatory or anti-allergy effects. The major components of the ethanol extracts of SC were: manassantin A, B, sauchinone (Quan et al., 2010; Min et al., 2009), and saucerneol D (Jung et al., 2011). Saucerneol D reduced inflammation and asthma changes by ameliorating oxydative stress. Manassantin is the phytochemical found in the methanol extract of Saururus chinensis roots and may control asthma and acute inflammation by inhibiting the generation of 5-lipoxygenase-dependent leukotriene C_4_ (Kim et al., 2011).

**Figure 2:**
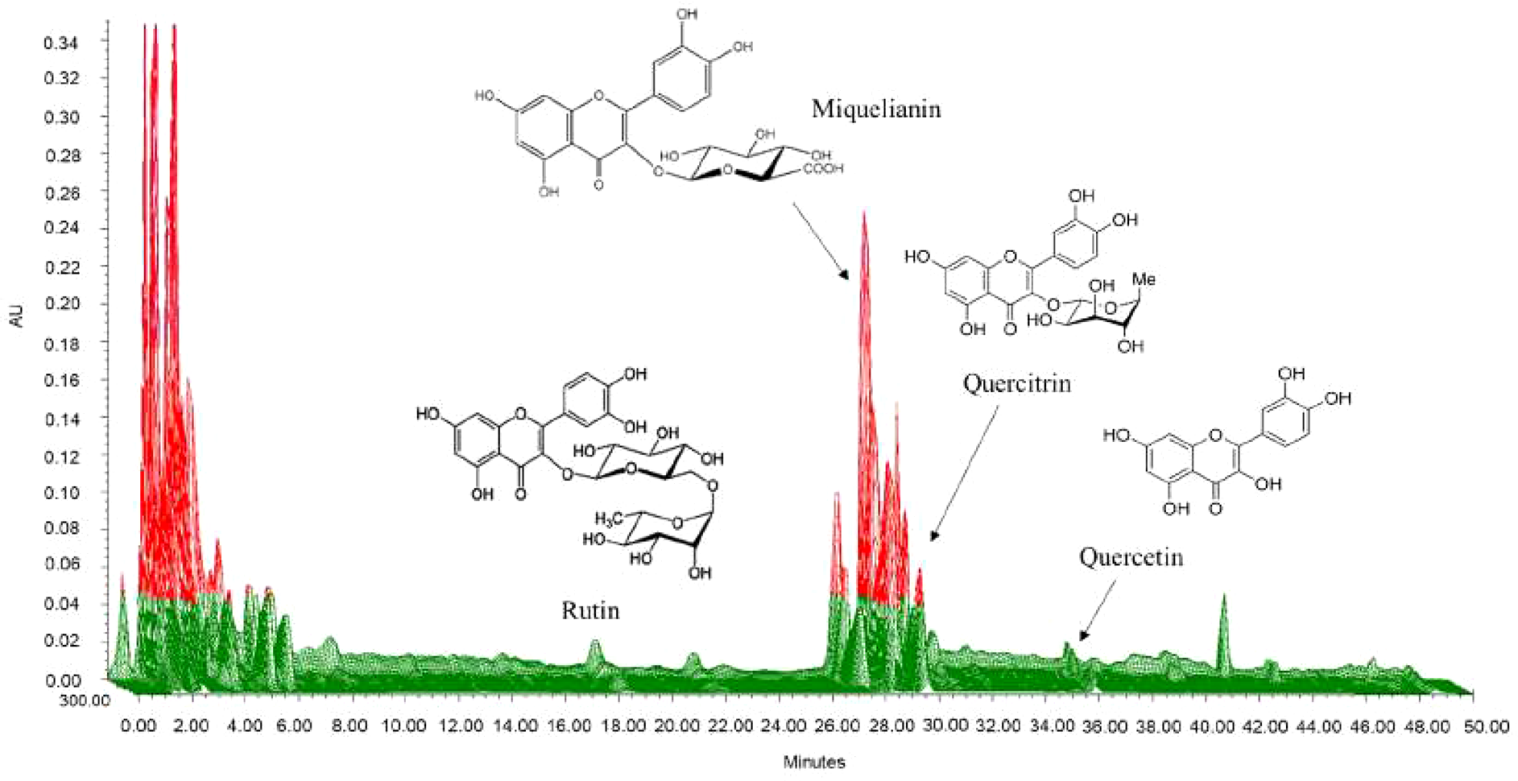
Three-dimensional high-performance liquid chromatogram of *Saururus chinensis* leaves extract. The retention time of rutin, miquelianin, and quercitrin were 18.3, 28.3 and 29.3 minute measured at 355 nm, 350 nm and that of quercetin was 35.8 minute measured at 365 nm.

We selected quercitrin, miquelianin, rutin, and quercetin as the standards by which to analyze the water extract of SC. We developed chromatographic conditions to optimize chromatograms with good resolution between the adjacent peaks. Rutin, miquelianin, quercitrin, and quercetin were eluted under optimized conditions at 18.3, 28.3, 29.3 and 35.8 min, respectively. The UV spectra were measured at 355 nm, 350 nm, and 365 nm, and the four standards were well absorbed (Fig. 2). The quality of the SC leaves was standardized to contain 11.75 mg/g miquelianin, 0.07 mg/g quercetin, 1.20 mg/g rutin and 2.38 mg/g quercitrin. Recent research has shown that the major components in SC are miquelianin, quercetin 3-*O*-(2″-*O*- β-glucopyranosyl)-α-rhamnopyranoside, and quercitrin (Nho et al., 2019). Miquelianin is the major consitutent of SC water-extract (11.75 ± 0.09 mg/g) and it has anti-inflammatory (Nho et al., 2019), anti-oxidative, anti-diabetic (Ahmed et al., 2019), and neuroprotective effects (Yuan et al., 2017). Rutin is one of the most well-known herbal constituents and is used therapeutically for the suppression of atopic dermatitis (Choi & Kim, 2013), and as an anti-asthmatic (Jung et al., 2007). Nho et al. (2019) reported that quercitrin is one of the major compounds in SC and may protect against anaphylactic shock (Cruz et al., 2008) and inhibit mucus secretions (Chang et al., 2010). The content of quercetin in SC was very low compared to other compoents (0.068 ± 0.017 mg/g) but some of its reported effects against pulmonary allergic disease include the relaxation of the smooth muscle of the airway (Townsend & Emala Sr, 2013) and an anti-asthmatic effect (Fortunato et al., 2012).

### SC suppressed the representative biological changes-related allergic pulmonary disease such as the number of white blood cells, neutrophils, and infammatory cells in BALF and IgE in serum

**Figure 3:**
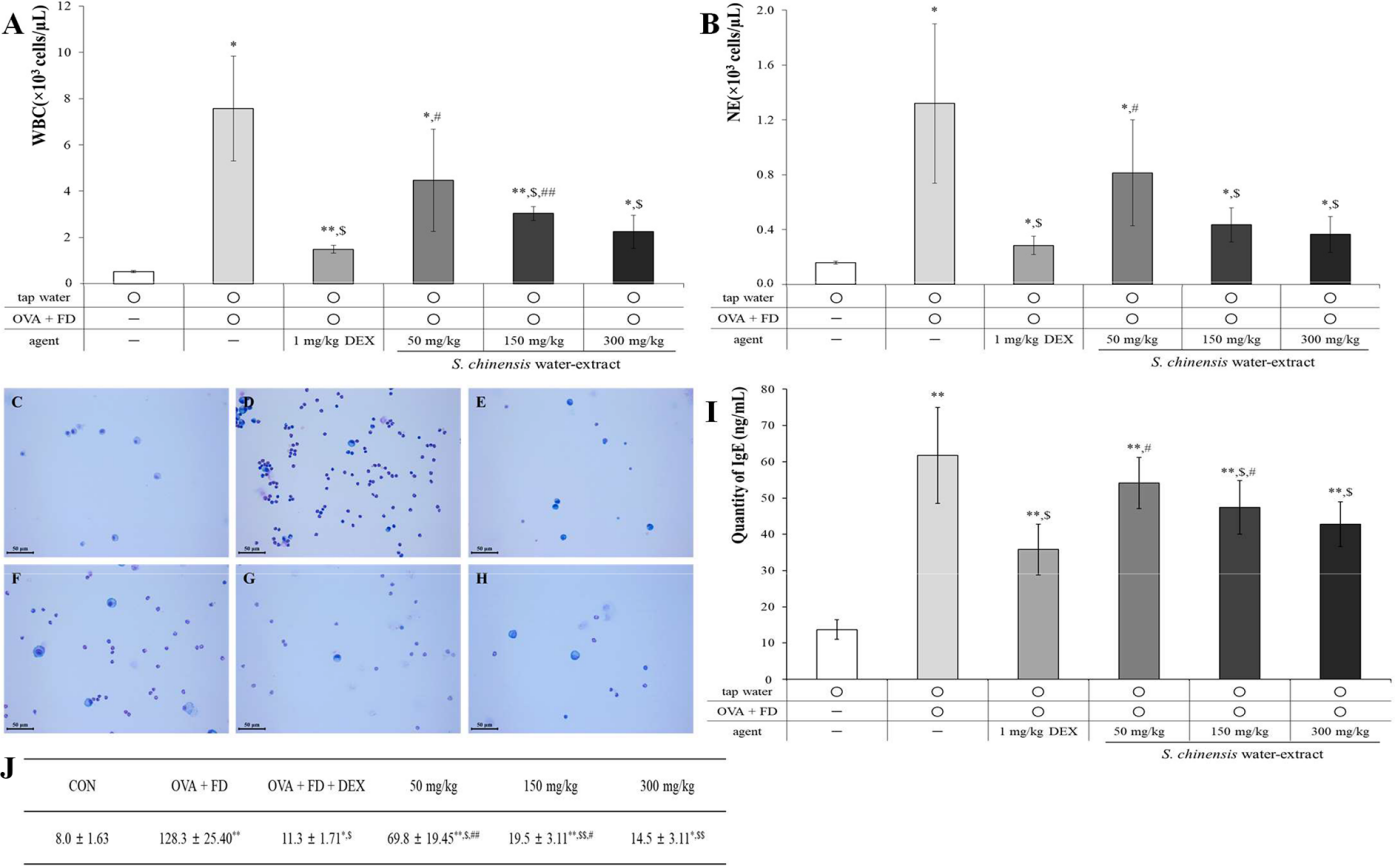
*Saururus chinensis* (SC) water-extract suppressed ovalbumin and find dust-induced allergic pulmonary disease. (A) SC dose-dependently down-regulated the number of white blood cell number in bronchioalveolar fluid (BALF) compared to that in ovalbumin and fine dust treatment group. (B) Among immune-related blood cells in BALF especially neutrophil (NE) was statistically suppressed by SC treatment. (C–H) In BALF the inflammatory cells number was dose-dependently inhibited by SC treatment. (I) SC effectively decreased the serum IgE expression and especially in 300 mg/kg SC treatment group, the level of IgE was similar to that in dexamethasone treatment group. (J) The number of inflammatory cells in BALF. C, tap water treatment (control) group; D, ovalbumin and fine dust treatment (induction) group; E, ovalbumin, fine dust, and dexamethasone treatment (positive control) group; F, ovalbumin, fine dust, and 50 mg/kg SC treatment group; G, ovalbumin, fine dust, and 150 mg/kg SC treatment group; H, ovalbumin, fine dust, and 300 mg/kg SC treatment group. WBC, white blood cell; NE, neutrophil; OVA, ovalbumin; FD, fine dust; DEX, dexamethasone. All values are presented as mean ± standard deviation. Scale bar, 50 µm. Magnification, ×400. *n* = 6 per each group. All values are presented as mean ± standard deviation. ^*^p < 0.05 *vs.* tap water treatment group; ^**^p < 0.001 *vs.* tap water treatment group; ^$^*p* < 0.05 *vs.* ovalbumin and fine dust treatment group; ^#^*p* < 0.05 *vs.* ovalbumin, fine dust and dexamethasone treatment; ^##^*p* < 0.001 *vs.* ovalbumin, fine dust and dexamethasone treatment.

Ovalbumin and fine dust induced the representative changes associated with allergic pulmonary disease compared to the effects of the tap water treatment (control) group (Fig. 3). The measurement of white blood cells (WBCs), neutrophils, and inflammatory cells in BALF is an important biological marker and the level of IgE in serum is known to increase in allergic reactions (Lee et al., 2019; Lee et al., 2017). SC dose-dependently suppressed the number of WBCs (Fig. 3A, *p* < 0.05) and neutrophils (Fig. 3B, *p* < 0.05) in BALF. The results in the 150 mg/kg SC treatment group and 300 mg/kg treatment group showed similar levels of WBCs and neutrophils to the dexamethasone treatment (positive control) group. The inflammatory cells in BALF were similar to the WBCs and neutrophils, and SC significantly and dose-dependently suppressed the number of inflammatory cells in BALF (Figs. 3C–3H). The results of IgE in SC treatment groups were similar to those of WBCs, neutrophils, and inflammatory cells, indicating that the SC treatment dose-dependently inhibited IgE expression, especially in the 150 mg/kg SC treatment and one level of IgE decreased to a consanguinity with that in the positive control (Fig. 3I, *p* < 0.05) 300 mg/kg treatment group. The 150 mg/kg SC treatment sufficiently inhibited the ovalbumin and fine dust-induced allergic pulmonary symptoms according to the results of BALF and serum testing. The suppression of allergic pulmonary symptoms may be related to the components of SC including miquelianin, rutin, quercitrin, and quercetin, according to the results of our study (Fig. 2).

### SC treatment controlled the representative morphological changes of allergic pulmonary disease

**Figure 4:**
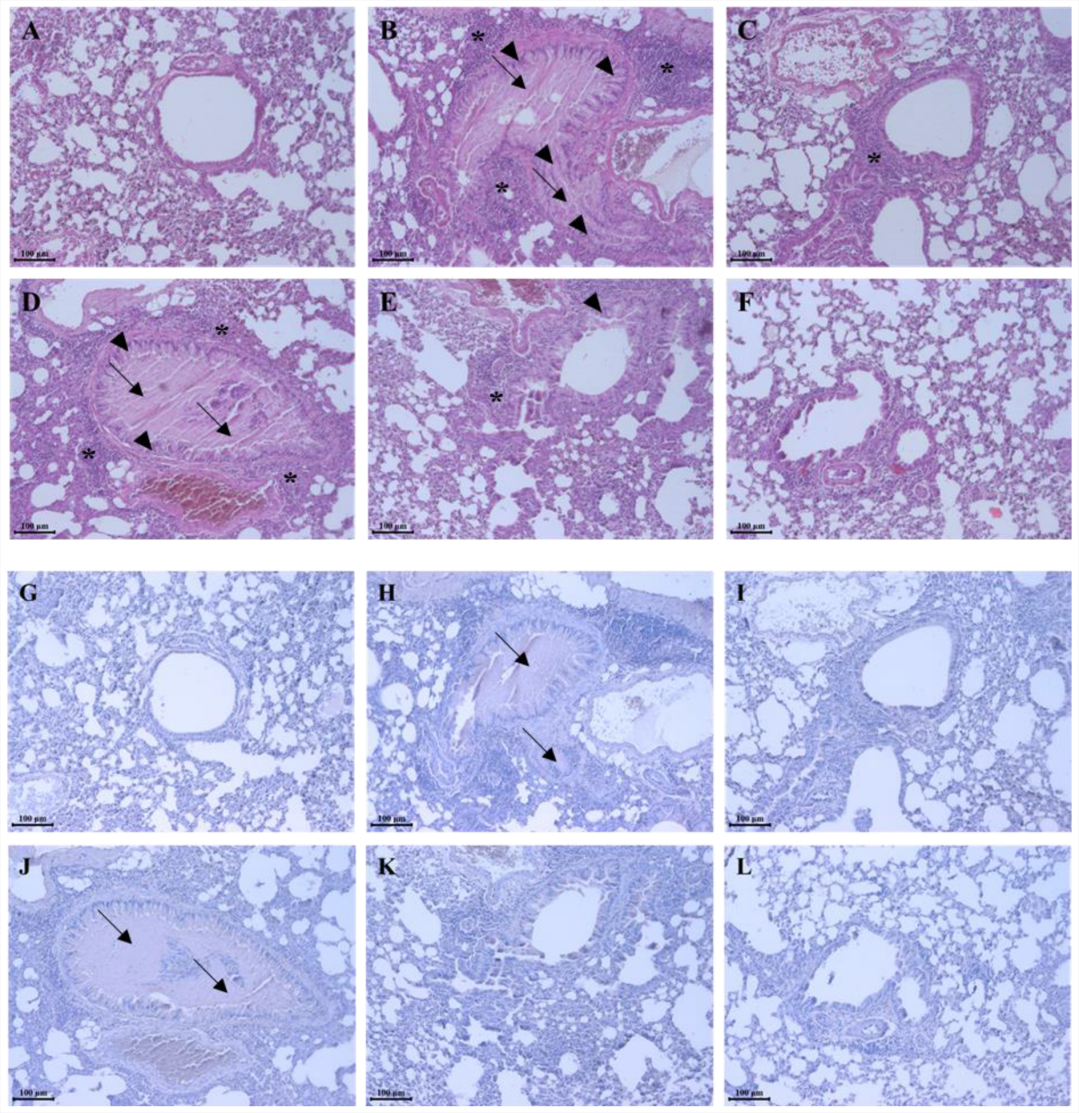
*Saururus chinensis* (SC) water-extract recovered the pulmonary morphological changes by ovalbumin and fine dust. (A–F) In order to compare the respiratory morphological changes H&E stain was conducted. SC dose-dependently controlled the ovalbumin and fine dust-induced morphological changes in the respiratory system such as mucous hypersecretion, pulmonary epithelial cell hyperplasia, and inflammatory cells infiltration. Especially in 300 mg/kg SC treatment group, the respiratory system was recovered more than in the positive control group. (G–L) To evaluate the accurate change of mucous in the bronchioalver duct PAS stain was done. Both in 150 mg/kg SC treatment group and in 300 mg/kg, one the mucous secretion was almost completely inhibited compared to the induction control group. A and G, tap water treatment (control) group; B and H, ovalbumin and fine dust treatment (induction) group; C and I, ovalbumin, fine dust, and dexamethasone treatment (positive control) group; D and J, ovalbumin, fine dust, and 50 mg/kg SC treatment group; E and K, ovalbumin, fine dust, and 150 mg/kg SC treatment group; F and L, ovalbumin, fine dust, and 300 mg/kg SC treatment group. Arrow, mucous hyper secretion; arrow head, pulmonary epithelial cell hyperplasia; asterisk, inflammatory cells infiltration. Scale bar, 100 µm. Magnification, ×200. *n* = 8 per each group.

Lung samples taken from test subjects were stained using hematoxylin & eosin (H&E) stain (Figs. 4A–4F & Table 2), and periodic acid Schiff (PAS) stain (Figs. 4G–4L) to evaluate the recovery effect of SC on the pulmonary morphological changes caused by ovalbumin and fine dust. In allergic pulmonary disease patients, typical pulmonary changes including mucous hypersecretion, pulmonary epithelial cell hyperplasia, and inflammatory cell infiltration near the bronchoalveolar duct and vessel were observed (Hirota & Martin, 2013; Fehrenbach, Wagner & Wegmann, 2017). A comparison of the morphology in tap water treatment group the pulmonary system to the representative morphological changes such as mucous hyper secretion (from 0.3 ± 0.45 to 2.7 ± 0.46), pulmonary epithelial cell hyperplasia (from 0.2 ± 0.45 to 2.4 ± 0.55), and inflammatory cells infiltration (from 0.3 ± 0.37 to 2.3 ± 0.45) were observed in the ovalbumin and fine dust treatment group (Figs. 4G–4L). Dexamethasone recovered the changes to tissues samples caused by ovalbumin and fine dust treatment (Fig. 4C) and SC dose-dependently and effectively controlled the morphological changes by ovalbumin and fine dust treatment (Figs. 4D–4F) including mucous hypersecretion (from 2.7 ± 0.46 to 0.6 ± 0.65), pulmonary epithelial cell hyperplasia (from 2.4 ± 0.55 to 0.7 ±0.67), and inflammatory cell infiltration (from 2.3 ± 0.45 to 0.6 ± 0.43). Ovalbumin and fine dust induced mucous hypersecretion, confirming the changes seen in the mucus PAS stain (Fig. 4H). Dexamethasone effectively controlled the mucous secretions (Fig. 4I) and SC dose-dependently down-regulated them (Figs. 4J–4L). Mucous secretions were almost completely inhibited in the 300 mg/kg SC treatment group (Fig. 4L). SC was shown to completely recover the morphological changes caused by ovalbumin and fine dust, which was similar to the results of the dexamethasone treatment. The controls effect against mucous secretions by SC may be caused by quercitrin, which is one of the constituents of SC (Fig. 2). Chang et al. (2010) reported that SC had an inhibitory effect on mucus secretions.

### SC effectively suppressed mRNA and protein levels of IL-13

IL-13 is an important cytokine related to allergic pulmonary disease. IL-13 causes abnormal physiological changes including inflammation, mucous hypersecretion, and fibrosis (Zhu et al., 1999). The mRNA and the protein levels of IL-13 were increased by ovalbumin and fine dust treatment (Fig. 5), and ovalbumin and fine dust treatments were controlled by dexamethasone treatment (*p* < 0.05). SC dose-dependently decreased the mRNA levels of IL-13 (*p* < 0.05, Fig. 5A) and the protein level of IL-13 was nearly controlled by a 50 mg/kg SC treatment (*p* < 0.05, Fig. 5B). The protein levels of IL-13 were similar to the dexamethasone treatment group in the 300 mg/kg SC treatment group. IL-13 is associated with various pulmonary allergic symptoms (Zhu et al., 1999), especially NF-κB activation (Chapoval et al., 2007). SC effectively controlled the IL-13 levels, which are known to stimulate inflammation and mucous hypersecretion. Thus, SC may suppress allergic pulmonary disease. Other cytokines related with allergic pulmonary disease, including IFN-γ, IL-5, and TNF-α, were evaluated and no change was shown in the SC (Fig. S1). Water extract of SC specifically controlled IL-13 compared to the ethanol extract of SC that suppressed various Th2-related cytokines, including IL-4, IL-5, and IL-13 (Quan et al., 2010; Min et al., 2009).

**Table 2:** The quantitative score chart of histopathological changes in the lung.

	Mucous hypersecretion (0–3)	Epithelial cell hyperplasia (0–3)	Inflammatory cell infiltration (0–3)
CON	0.3 ± 0.45	0.2 ± 0.45	0.3 ± 0.37
OVA + FD	2.7 ± 0.46^**^	2.4 ± 0.55^**^	2.3 ± 0.45^**^
OVA + FD + DEX	0.7 ± 0.44^#^	0.9 ± 0.55^$^	0.9 ± 0.56^$^
50 mg/kg *Saururus chinensis*	2.7 ± 0.45^**^,^##^	2.6 ± 0.46^**^,^##^	2.5 ± 0.50^**^,^#^
150 mg/kg *Saururus chinensis*	1.8 ± 0.48^**^,^$#^	1.7 ± 0.49^**^,^#^	1.3 ± 0.45^*^,^$^
300 mg/kg *Saururus chinensis*	0.6 ± 0.65^#^	0.7 ± 0.67^$^	0.6 ± 0.43^#^

**Notes.**

OVA ovalbumin FD fine dust DEX dexamethasone

* *p* < 0.05 vs. tap water treatment group.

** *p* < 0.001 vs. tap water treatment group.

$ *p* < 0.05 vs. ovalbumin and fine dust treatment group.

# *p* < 0.05 vs. ovalbumin, fine dust and dexamethasone treatment.

## *p* < 0.001 vs. ovalbumin, fine dust and dexamethasone treatment.

**Figure 5:**
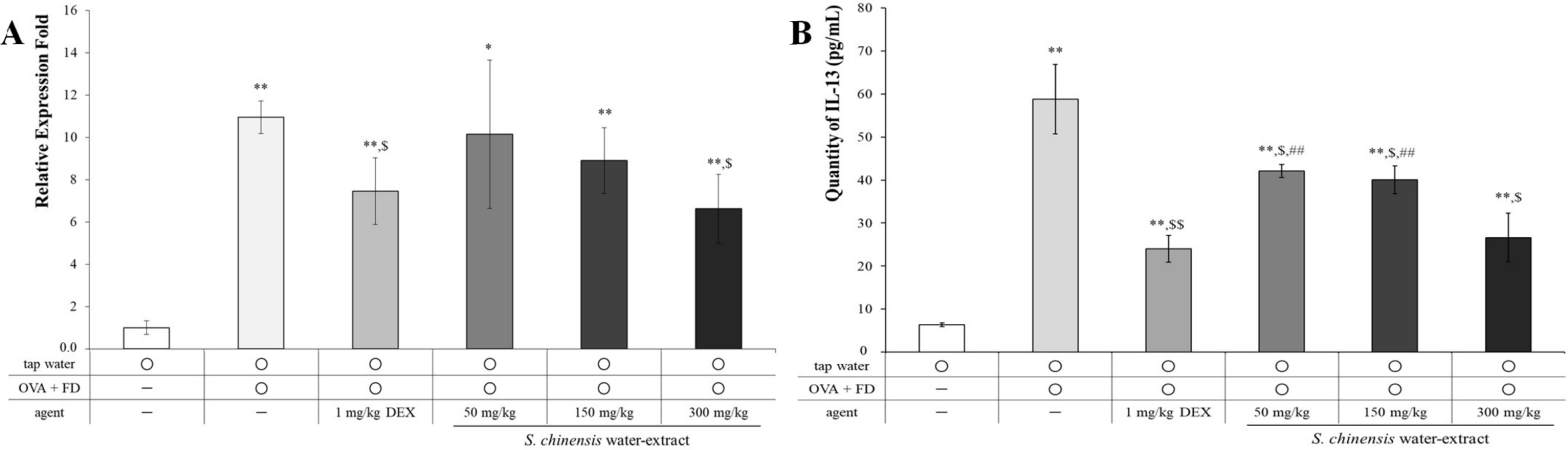
*Saururus chinensis* (SC) water-extract dose-dependently inhibited mRNA level (A) and the protein level (B) of IL-13 in lung lysates. The relative expression level of mRNA level of IL-13, which was increased by ovalbumin and fine dust treatment was dose-dependently decreased by SC treatment. Especially in 300 mg/kg SC treatment, the mRNA level of IL-13 was down-regulated similar to that in dexamethasone treatment. The expression level of IL-13 protein was completely controlled by SC treatment although in 50 mg/kg SC treatment the level of was similar to that in dexamethasone treatment group. OVA, ovalbumin; FD, fine dust; DEX, dexamethasone. All values are presented as mean ±standard deviation. *n* = 8 per each group. ^*^p < 0.05 *vs.* tap water treatment group; ^**^p < 0.001 *vs.* tap water treatment group; ^$^*p* < 0.05 *vs.* ovalbumin and fine dust treatment group; ^##^*p* < 0.001 *vs.* ovalbumin, fine dust and dexamethasone treatment.

### SC completely inhibited the NF-κB translocation and COX-2 protein expression and then suppressed the level of PGE_2_

**Figure 6:**
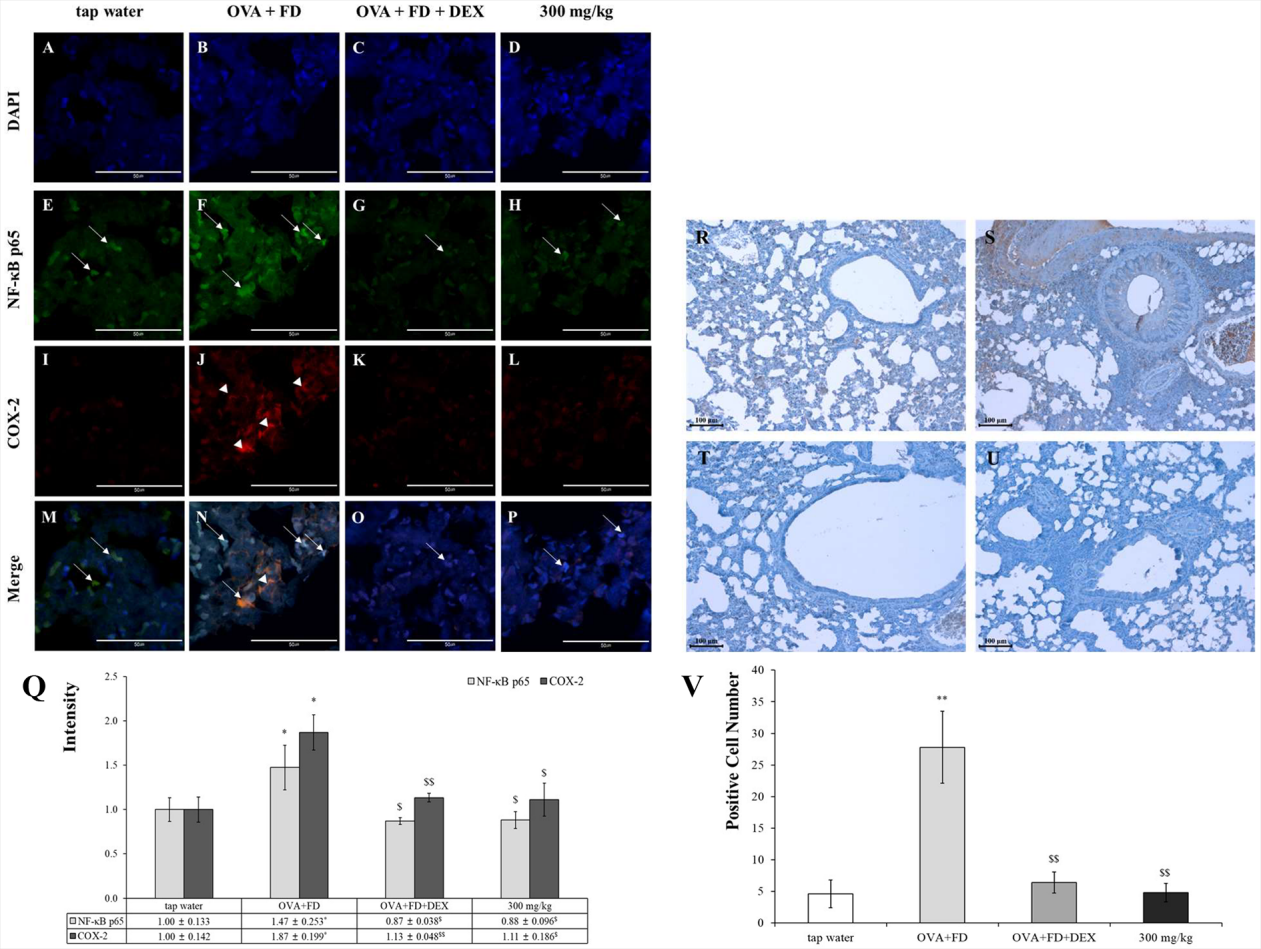
*Saururus chinensis* (SC) water-extract controlled inflammation through the NF-κ B/COX-2 and PGE_2_ axis. (A–P) *Saururus chinensis* (SC) water-extract inhibited COX-2 expression via preventing NF-κ B translocation. Compared to the results in ovalbumin and fine dust treatment group in tap water treatment group the expression of COX-2 protein (red spot) was decreased significantly. In ovalbumin and fine dust treatment group NF-κ B proteins were translocated from cytoplasm to nucleus and the expression of COX-2 protein was increased effectively. Both in dexamethasone treatment group and in 300 mg/kg SC treatment group, the proteins expression were down-regulated, both of NF-κ B and COX-2. (R–I) SC effectively inhibited the PGE_2_ expression. R, tap water treatment (control) group; S, ovalbumin and fine dust treatment (induction) group; T, ovalbumin, fine dust, and dexamethasone treatment (positive control) group; I, ovalbumin, fine dust, and 300 mg/kg SC treatment group. Arrow, NF-κ B; arrowhead, COX-2. Magnification, 1000 ×. Scale bar, 50 *μ*m. (Q) The quantitative score of NF- *κ* B p65 vs. nucleus or COX-2 vs. nucleus. All the values are presented as mean ± standard deviation. *n* = 8 per each group. ^*^p < 0.05 *vs.* tap water treatment group; ^$^*p* < 0.05 *vs.* ovalbumin and fine dust treatment group; ^$$^*p* < 0.001 *vs.* ovalbumin and fine dust treatment group. (V) The number of PGE_2_ positive cells in the lung tissue. All values are presented as mean ± standard deviation. ^**^p < 0.001 *vs.* tap water treatment group; ^**^p < 0.001 *vs.* tap water treatment group; ^$$^*p* < 0.001 *vs.* ovalbumin and fine dust treatment group.

Ovalbumin and fine dust treatments increased the NF-κB levels in the nucleus and the COX-2 protein in the cytoplasm but dexamethasone down-regulated their levels (Figs. 6A–6Q). The expression of the COX-2 protein was almost visible in the tap water treatment group. The COX-2 expression levels were similar in the tap water treatment group, the dexamethasone treatment group, and the SC treatment group. The expression levels of PGE_2_ were similar to those of NF-κB and COX-2 (Figs. 6R−6V). The NF-κB/COX-2 and PGE_2_ axes are significant pathways; PGE_2_ was released by NF-κB translocation from the cytoplasm to the nucleus and COX-2 protein were synthesized in the cytoplasm. PGE_2_ is a very important biomarker for inflammation (Mishra, Banga & Silveyra, 2018a; Mishra, Banga & Silveyra, 2018b).

SC has been used as a traditional medicine in the northeastern regions of Asia for generations without reports of toxicity. There are many medicinal herbs that show no adverse effects and are relatively safe when compared to the effects of pharmaceutical drugs used to suppress allergic pulmonary diseases.

## Conclusions

We confirmed the extracts from SC water extract, specifically miquelianin, rutin, quercitrin, and quercetin (LHF618^^®^^), effectively controlled pulmonary allergic symptoms induced by ovalbumin and fine dust. SC water extract improved symptoms by decreasing white blood cells, reducing the presence of neutrophils in bronchioalveolar fluid and IgE in serum, recovering the morphological changes of mucous hypersecretion, improving pulmonary epithelial cell hyperplasia and inflammatory cell infiltration, down-regulating mRNA and protein levels of IL-13 and their inflammatory pathways, participating in NF-κB translocation/COX-2 protein synthesis, and improving the level of PGE_2_. Allergic pulmonary disease may have a number of triggers and may include chronic inflammation. Our results verified that the control activity of SC against allergic pulmonary disease was based on the NF-κB/COX-2 and PGE_2_ anti-inflammatory pathways. Additional studies should work to develop SC as a drug candidate against fine dust-induced allergic pulmonary disease.

##  Supplemental Information

10.7717/peerj.10043/supp-1 Supplemental Information 1 The results of IFN-γ ’s mRNA level (A) and protein level (B), IL-5’s mRNA level (C) and protein level (D), and TNF- α’s mRNA level (E) and protein level (F) in lung lysates ^*^p < 0.05 *vs.* tap water treatment group; ^**^p < 0.001 *vs.* tap water treatment group; ^$^*p* < 0.05 *vs.* ovalbumin and fine dust treatment group; ^#^*p* < 0.05 *vs.* ovalbumin, fine dust and dexamethasone treatment.

10.7717/peerj.10043/supp-2 Supplemental Information 2 Raw data for Table 3 and Figs. 2, 4 and 5

## Additional Information and Declarations

### Data Availability

The following information was supplied regarding data availability: The raw measurements are available in the Supplementary File.
