# Supplementary figures and images for: Saururus chinensis-controlled allergic pulmonary disease through NF-κB/COX-2 and PGE2 pathways
Source: PeerJ. 2020 Sep 24;8:e10043. doi: 10.7717/peerj.10043 (PMC7520084; doi:10.7717/peerj.10043)

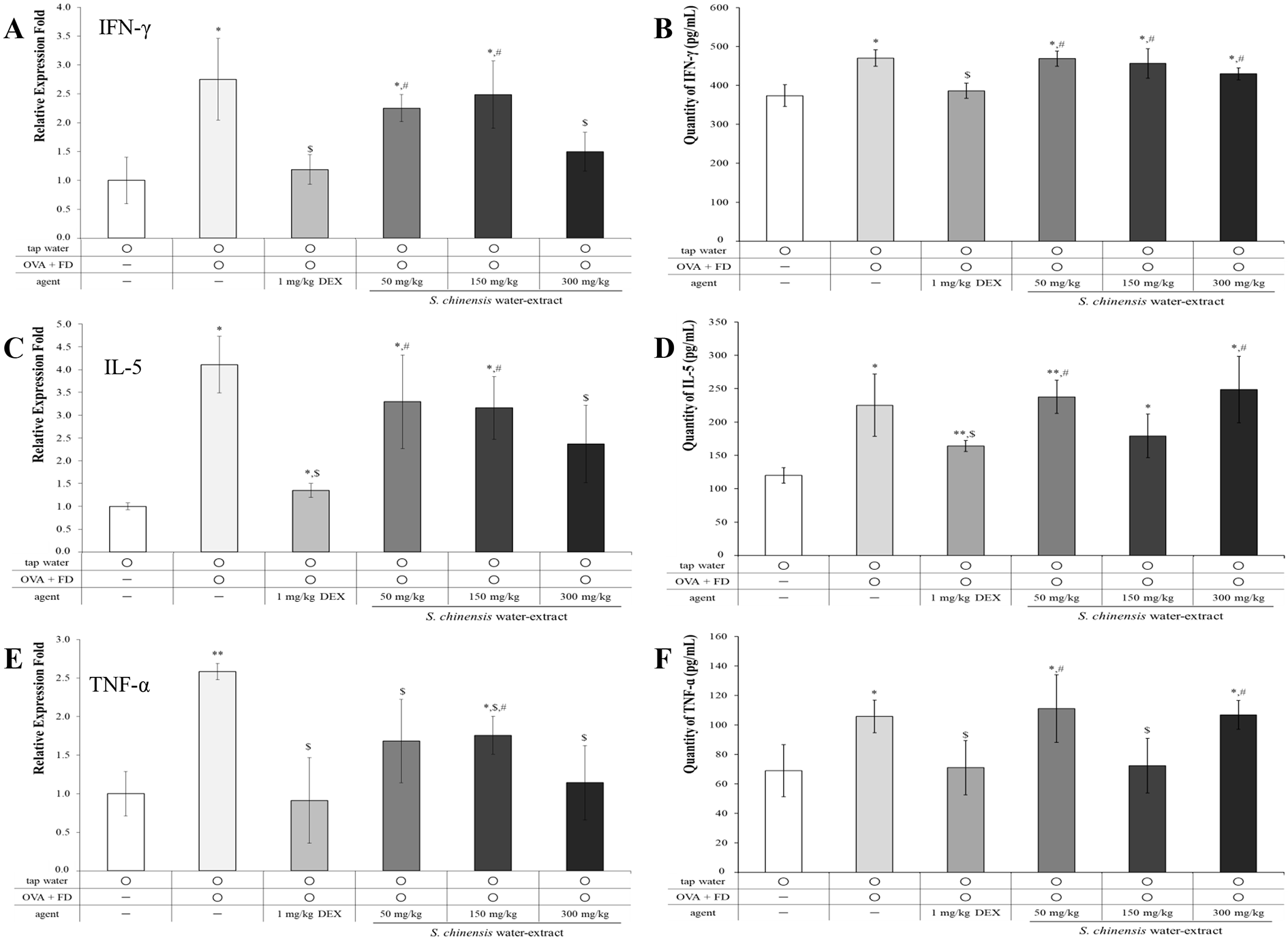

Supplement: Supplemental Information 1 — *p < 0.05 vs. tap water treatment group; **p < 0.001 vs. tap water treatment group; $p < 0.05 vs. ovalbumin and fine dust treatment group; #p < 0.05 vs. ovalbumin, fine dust and dexamethasone treatment. [file peerj-08-10043-s001.png]
